# Association Analysis of Genetic Variants with Type 2 Diabetes in a Mongolian Population in China

**DOI:** 10.1155/2015/613236

**Published:** 2015-07-28

**Authors:** Haihua Bai, Haiping Liu, Suyalatu Suyalatu, Xiaosen Guo, Shandan Chu, Ying Chen, Tianming Lan, Burenbatu Borjigin, Yuriy L. Orlov, Olga L. Posukh, Xiuqin Yang, Guilan Guilan, Ludmila P. Osipova, Qizhu Wu, Narisu Narisu

**Affiliations:** ^1^Inner Mongolia University for the Nationalities, Tongliao, Inner Mongolia 028000, China; ^2^BGI-Shenzhen, Shenzhen 518083, China; ^3^Department of Biology, University of Copenhagen, 2100 Copenhagen, Denmark; ^4^The Institute of Cytology and Genetics, Siberian Branch of the Russian Academy of Sciences (SB RAS), Novosibirsk 630090, Russia; ^5^Novosibirsk State University, Novosibirsk 630090, Russia; ^6^College of Animal Science and Technology, Northeast Agricultural University, Harbin 150030, China; ^7^Medical Genomics and Metabolic Genetics Branch, National Human Genome Research Institute, National Institutes of Health, Bethesda, MD, USA

## Abstract

The large scale genome wide association studies (GWAS) have identified approximately 80 single nucleotide polymorphisms (SNPs) conferring susceptibility to type 2 diabetes (T2D). However, most of these loci have not been replicated in diverse populations and much genetic heterogeneity has been observed across ethnic groups. We tested 28 SNPs previously found to be associated with T2D by GWAS in a Mongolian sample of Northern China (497 diagnosed with T2D and 469 controls) for association with T2D and diabetes related quantitative traits. We replicated T2D association of 11 SNPs, namely, rs7578326 (*IRS1*), rs1531343 (*HMGA2*), rs8042680 (*PRC1*), rs7578597 (*THADA*), rs1333051 (*CDKN2*), rs6723108 (*TMEM163*), rs163182 and rs2237897 (*KCNQ1*), rs1387153 (*MTNR1B*), rs243021 (*BCL11A*), and rs10229583 (*PAX4*) in our sample. Further, we showed that risk allele of the strongest T2D associated SNP in our sample, rs757832 (*IRS1*), is associated with increased level of TG. We observed substantial difference of T2D risk allele frequency between the Mongolian sample and the 1000G Caucasian sample for a few SNPs, including rs6723108 (*TMEM163*) whose risk allele reaches near fixation in the Mongolian sample. Further study of genetic architecture of these variants in susceptibility of T2D is needed to understand the role of these variants in heterogeneous populations.

## 1. Introduction

Type 2 diabetes (T2D) is a complex disease characterized by insulin resistance and pancreatic beta-cell dysfunction. In China, 9.7% and 15.5% of the entire population suffer from T2D and prediabetes, respectively [1]. Given recent advances in genotyping and sequencing technology, the GWAS have contributed significantly to the identification of susceptibility loci for T2D and many other complex disorders. At least 80 loci conferring susceptibility to T2D have been identified to date [2, 3] (http://www.genome.gov/gwastudies/) and the genetic architecture underlying T2D varies substantially between populations of different ethnic backgrounds [4, 5]. Studying genetics of T2D in multiethnic cohorts has been insightful for fine-mapping casual variants and identifying new loci [6], demonstrating the use of investigating common variants in different ethnic samples.

There are 10 million Mongolians currently living in various regions of Asia [7]. To our knowledge, there is only one T2D association study conducted in a Mongolian sample, which replicated association of variants in* KCNQ1* and* ABCC8* with T2D [8]. However, the small-sized study (177 cases and 216 controls) has low power to detect variants with smaller effect. The prevalence of T2D in Mongolians in China has grown among the adult urban population from 1.86% in 1980 to 5.6% in 2012 [9–11]. In our study, we aim to explore genetic risks of 34 T2D SNPs previously reported by GWAS in a larger Mongolian sample. Twenty-eight SNPs passed rigorous quality control filtering and 11 of them showed significant association with T2D (Bonferroni corrected *P* < 0.05). The SNP with the strongest T2D association, rs7578326, has the risk allele significantly associated with increased levels of TG. We demonstrated the need of further study of allelic difference of T2D associated SNPs in diverse populations.

## 2. Methods and Materials

### 2.1. Ethics Statement

This study was approved by the Institutional Review Board of the Affiliated Hospital of Inner Mongolia University for the Nationalities and complied with the Declaration of Helsinki. The written informed consent was obtained from each participant.

### 2.2. Study Population

We collected whole blood samples from 986 individuals of Mongolian ethnicity from Inner Mongolia, China. The sample was comprised of 511 T2D cases and 475 healthy normoglycemic controls, of which 497 cases and 469 controls passed quality control filtering and were used for subsequent analysis (see below). Cases were registered based on the World Health Organization (WHO) criteria [12] of fasting plasma glucose concentration ≥7 mmol/L or 2-h plasma glucose concentration ≥11.1 mmol/L and were admitted to the affiliated hospital of the Inner Mongolia University for Nationalities. Nondiabetic healthy controls were selected based on matching sex and ethnic background from the same region. Aside from the diagnosis of T2D, we collected other diabetes related lipid traits, such as TC, HDL-C, LDL-C, and TG, for each individual. We collected certain life style information (smoking and drinking habits), waist circumference (WC), and body mass index (BMI) of each participant as well.

### 2.3. Selection of SNPs and Genotyping

We selected a list of SNPs previously found to be associated with T2D based on the NHGRI GWAS catalog [2] (available at http://www.genome.gov/gwastudies/, November, 2012). Candidate SNPs were initially selected with the following considerations: (1) SNPs found to be associated with T2D in an Asian sample were given higher priority (rs6723108 and rs5945326 were added after the initial selection date); and (2) subsequently SNPs found to be associated with multiple studies were included. We were able to genotype 34 SNPs located in or near 33 candidate genes (see Supplementary Material available online at http://dx.doi.org/10.1155/2015/613236). We included two SNPs around* KCNQ1*, because those have been reported to be associated with Asian populations [13, 14].

We estimated the concentration of isolated genomic DNA using Qubit dsDNA BR Assay Kit (Invitrogen, USA), and the DNA solution was further diluted to a concentration of 10 ng/*μ*L. We designed the targeted sequencing primers and redesigned the primer sets with dispersed or weak electrophoretic bands. To prepare the chip array, we used a multisample nanodispenser (WaferGen, USA) to disperse DNA and primers into SmartChip MyDesign Chip (WaferGen, USA). Following the polymerase chain reaction (PCR) amplification, we purified PCR products through Agencourt AMPure XP-medium beads to get mixed Illumina pair-end libraries. Insert sizes were calculated by Aglient 2100 bioanalyzer (Agilent, USA) and concentrations were estimated by Real Time PCR. Sequencing was performed on either Illumina MiSeq or on Illumina Hiseq 2500. All sequencing steps were in strict accordance with Illumina recommended protocols.

The final sequencing depth reached > 200x, and the length of pair-end reads was 100 bp. Reads with an average base quality of ≥20 were kept for further analysis. BWA [15] (v0.5.9, available at http://bio-bwa.sourceforge.net/) was used to map all clean reads against the human reference genome of hg19 allowing ≤3 mismatches across a single read. Samtools mpileup (v0.1.18, available at http://samtools.sourceforge.net/) command was used to obtain SNP genotypes as described in [16].

These genotypes were further filtered according to the following criteria: SNPs with ≥5% of missing call rate across the samples. Samples with ≥3% of missing genotypes (which corresponds to 10% of missing SNP call rate) were removed. We tested SNPs for Hardy-Weinberg Equilibrium (HWE) and excluded SNPs with HWE *P* value < 1 × 10^−6^ in unaffected individuals. Twenty-eight SNPs of 966 samples (497 cases and 469 controls) passed the quality control filtering, and the overall genotype call rate is 99.3% or higher across the sample.

### 2.4. Statistical Analysis

We tested association between candidate SNPs and the status of T2D using logistic regression (likelihood ratio test) by adjusting for the effects of age, sex, and BMI. The study-wide significance was determined by applying Bonferroni correction using 28 tested SNPs (*P* value ≤ 0.05/28 = 1.8 × 10^−3^). We tested association with diabetes related quantitative traits (TC, HDL-C, LDL-C, and TG) across both T2D cases and controls using linear regression with the age, sex, BMI, and T2D status as covariates. All quantitative trait measures were normalized by quantile normalization and the normalized values were used in the analyses. Formal statistical tests, including 95% confidence intervals (CI), were performed using EPACTS [17] (v3.2.6, available at http://www.sph.umich.edu/csg/kang/epacts/). Differences in population structure between the Mongolian sample (healthy controls) and healthy Caucasian (CEU) or Chinese (CHB and CHS) samples of 1000 G project [18] (http://www.1000genomes.org/) were estimated by comparing risk allele frequency and the Wright's fixation index (*F*_ST_) using plink [19, 20]. Comparison of trait values between cases and controls was conducted using Student's *t*-test.

## 3. Results

After rigorous sample and marker level quality control filtering, genotypes of 28 SNPs on 966 individuals (including 497 with T2D cases and 469 nondiabetic ethnically matched controls) were kept for subsequent analyses. Clinical characteristics of the sample are summarized in Table 1. Overall, consistent with previous studies [21], T2D cases in current study have higher TC, TG, and LDL-C values compared to controls and have comparable HDL-C values with the controls, indicating that TC, TG, and LDL-C are among risk factors for T2D in the Mongolian sample.

Table 2 presents the association results between the 28 SNPs and T2D status. Of the 28 SNPs tested, 11 SNPs were significantly associated after correcting for multiple testing (*P* < 1.8 × 10^−3^). We replicated a T2D association near* KCNQ1 *(rs2237897; OR = 1.39; *P* = 0.002), originally identified in a Japanese population [14], and subsequently replicated in another Mongolian population sample with the same ethnic background as our sample [22]. We also replicated T2D association of three SNPs initially identified in Asian samples, namely, rs163182 (*KCNQ1*) [13] in Japanese, rs6723108 (*TMEM163*) [23] in Indians, and rs10229583 (*PAX4*) [24] in Chinese samples. In addition, we replicated associations of seven T2D SNPs previously identified in European populations. To our knowledge, the association of rs7578326 (*IRS1*), rs1531343 (*HMGA2*), rs8042680 (*PRC1*), rs1387153 (*MTNR1B*), rs7578597 (*THADA*) [25], rs243021 (*BCL11A*) [26], and rs1333051 (*CDKN2*) [27] was for the first time replicated in an Asian sample.

**Figure 1:**
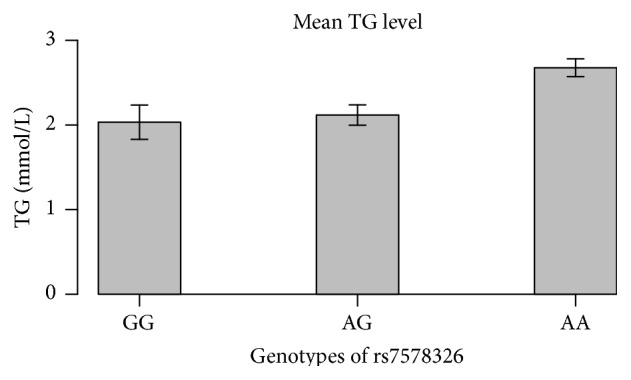
Correlation of mean triglyceride level with the genotypes of rs7578326 in the Mongolian sample. *y*-axis represents TG (mmol/L) level, and *x*-axis represents samples with different genotypes. Error bar represents standard error of TG in the samples with the specific rs7578326 genotype. Number of samples with genotypes GG, AG, and AA are 47, 248, and 647, respectively, across all samples including T2D cases and controls.

Among the four lipid related traits tested, we only observed a single significant association of T2D risk allele A of rs7578326 (*IRS1*) with TG level (*P* = 0.0004, OR = 3.4, and CI 1.7–6.6). Mean TG level for AA, AG, and GG is 2.65, 2.12, and 2.03 mmol/L (Figure 1). Individuals homozygous for the risk allele A have 25% higher TG values compared to heterozygotes. Heterozygotes have 4% higher TG compared to those homozygous for the nonrisk allele G.

Most of the SNPs selected for testing in our sample have been identified to be associated with T2D initially in European populations. The lack of replication of 17 T2D loci identified by the GWAS studies in other populations prompted us to look at the differences of allelic architecture in the SNPs tested for the T2D association in our population. We observed variable risk allele frequency difference between our sample and 1000 G Caucasian (CEU) and Chinese populations (Table 3). Six out of 11 SNPs that have risk allele frequency 10% higher in the Mongolian sample compared to the Caucasian sample in 1000 G panel showed significant association with T2D in our study, and only two out of nine SNPs that have 10% or lower risk allele frequency compared to CEU showed significant association with T2D. Although statistically not significant, this observation shows a trend of overabundance of T2D associated SNPs in those with high frequency of risk alleles in the Mongolian population. In addition, we calculated Wright's fixation index to estimate whether the heterogeneity of a tested SNP is different between the Mongolian sample and Caucasian or Chinese samples. Noteworthy, a T2D associated SNP, rs6723108 (*TMEM163*), has reached near fixed high risk allele frequency (0.98 in our sample compared to 0.51 in the Caucasian sample of 1000 G project) and has a substantial population difference with the Caucasian (*F*_ST_ as high as 0.61). This trend is also present for the case of rs8042680 (PRC1), which has much higher risk allele frequency in the Mongolian sample compared to the Caucasians (0.92 versus 0.72; *F*_ST_ = 0.67). Although it is difficult to postulate the cause of the population difference, high proportion of Mongolians appear to carry this risk allele.

## 4. Discussion

In this study, we chose to examine the association of 34 GWAS SNPs, previously identified in European and East Asian populations, with susceptibility to T2D in a Mongolian sample from China. Six SNPs did not pass quality control filtering and were excluded from the analysis.

This study confirmed the T2D association of rs2237897 in* KCNQ1 *that were reported in European, Mexican, Chinese, Japanese, and Mongolian populations [8]. We also replicated the T2D association of three SNPs previously identified in Asian samples, namely, rs163182 (*KCNQ1*) [13] in Japanese, rs6723108 (*TMEM163*) [23] in Indians, and rs10229583 (*PAX4*) [24] in Chinese samples. In addition, our study replicated T2D association of seven additional GWAS SNPs in our sample. To our knowledge, the T2D association of rs7578326 (*IRS1*), rs1531343 (*HMGA2*), rs8042680 (*PRC1*), rs1387153 (*MTNR1B*), rs7578597 (*THADA*) [25], rs243021 (*BCL11A*) [26], and rs1333051 (*CDKN2*) [27] was replicated in an East Asian sample for the first time. This indicates that our study was able to replicate the result obtained in a population with the similar ethnic background and extended the replication of several other loci in an Asian population.

Although the association is not significant after the multiple test correction, additional six SNPs show the OR trend consistent with the original reported studies. SNPs found to be associated with T2D in Asian populations, rs1048886 (*C6orf57*) [5], rs4402960 (*IGF2BP2*) [28], rs5015480 (*HHEX, IDE*), rs1359790 (*SPRY2*) [29], rs1552224 (*CENTD2*), rs3923113 (*GRB14*), rs5215 (*KCNJ11*), rs7903146 (*TCF7L2*) [6], rs10886471 (*GRK5*), and rs7403531 (*RASGRP1*) [30], were not replicated in our study. This observation clearly suggests the following: (1) our study has a limited power because of relatively small sample size, which warrants further confirmation of these SNPs in a larger sample from the same ethnic group; (2) our sample has variable degrees of difference in risk allele frequency compared to Caucasian population where most original GWAS were conducted, and (3) the selection of control individuals was not matched precisely with the cases, in particular, with respect to age, and there is a trend that the healthy controls are younger compared to the cases. However, we took a step to use age, sex, and BMI as covariates in our statistical analysis to minimize the effect of this disparity. The follow-up larger scale study should recruit more matching control individuals to cases.

None of the SNPs we tested for association with T2D here has been previously implicated to be associated with lipids based on the NHGRI GWAS catalog (available at http://www.genome.gov/gwastudies/, November, 2012). However, we observed that association of T2D risk allele (A, major allele) of rs7578326 (*IRS1)*   is associated with the increased level of TG. An elevated level of TG has been implicated as a risk factor of T2D, which likely resulted from the diminished activity of insulin causing inhibition of microsomal TG transfer protein activity [31]. The major allele (A) of a SNP in the upstream region of the same gene (*IRS1*), namely, rs2972146, is reported to be associated with an elevated level of TG as well [32]. rs297214 has nominal linkage disequilibrium with the SNP reported here (*r* ^2^ = 0.3753, 1000 G phase I), indicating a possibility that the T2D associated SNP or its proxy could be playing a role in pathogenesis of T2D or its related TG metabolism through* IRS1* activity. Further functional work will help to understand the role of this SNP. Since we did not have information on previous medical history of treatment for either high cholesterol or T2D for the patients, it is possible that such treatments, if administered previously, could have prevented us from seeing the effect of SNP association with the diabetes related quantitative traits, including TG.

Mongolians are one of the people who reside on the Mongolian Plateau in Asia and have heavily depended on nomadic life styles with harsh environments characterized by low temperature and scarce availability of food sources [33]. Although we do not have any concrete evidence that these variants might play a role in conserving energy, we found six T2D associated SNPs in our sample that have 10% or higher risk allele frequency compared to Caucasian samples. These SNPs have a comparable risk allele frequency with Chinese populations in the 1000 G project, indicating that our allele frequency estimate is reflecting the allelic structure of the SNPs in the populations of Asia. We observed that SNPs rs6723108 (*TMEM163*) and rs8042680 (*PRC1*) have substantial allelic differences in our sample compared to Caucasians and the risk alleles have reached near fixed level in Mongolians. On the other hand, a widely replicated T2D SNP, rs7903146 (*TCF7L2*), in European populations has substantially low risk allele frequency in the Mongolian sample (0.06 versus 0.31 in the Caucasian sample; *F*_ST_ = 0.27) and is not associated in our sample of modest size. It is likely that the frequency difference of some SNPs between our sample and European population also contributed to the lack of reproducibility in T2D association in our study.

Although we note that rs6723108 has a wide range of OR estimate (95% CI 2–33.3; risk allele frequency 0.98), the OR of 7.7 in our study is substantially greater than what was reported in the original study in an Indian population [23] (OR = 1.31, 95% CI 1.20–1.44; risk allele frequency 0.89), indicating the potential higher effect of the variant in our sample. More systematic studies of population specific allelic architecture with respect to T2D are needed to dissect the potential impact of these highly differentiated SNPs in different populations.

In conclusion, our association study has confirmed association of several previously identified T2D susceptibility loci in the Mongolian sample. We also identified rs7578326 near* IRS1* to be associated with an increased level of TG. The observation of the remarkable allele frequency difference of the T2D SNPs in our sample compared to Caucasians is important in further identifying causative variants for T2D and understanding the role of these SNPs in development of T2D in different ethnic populations.

## Supplementary Material

The SNPs were chosen with following considerations: (1) GWAS SNPs found to be associated with T2D in an Asian sample were given higher priority; and (2) subsequently, SNPs found to be associated with T2D in multiple studies were included. Genotyping of six SNPs was not successful, thus these SNPs were included from further analysis.

## Figures and Tables

**Table 1:** Clinical characteristics of study population.

Traits	Controls^a^	Cases	*P* value for normalized trait value^b^
Samples (*N*)	469	497	
Sex (male/female)	168/301	214/283	
Smoking (%)	22.2	31.6	
Drinking (%)	38.6	27.2	
Age (years)	40.5 ± 15.8	54.3 ± 10.1	
BMI (kg/m^2^)	24.0 ± 4.8	26.1 ± 4.0	<0.001
WC (cm)	81.7 ± 14.5	92.0 ± 11.6	<0.001
TC (mmol/L)	4.6 ± 1.2	5.4 ± 1.2	<0.001
TG (mmol/L)	1.9 ± 1.5	3.1 ± 2.9	<0.001
HDL-C (mmol/L)	1.3 ± 0.3	1.4 ± 1.0	0.775
LDL-C (mmol/L)	2.7 ± 0.9	3.2 ± 1.0	<0.001

^a^Mean ± SD.

^b^Student's *t*-test. BMI: body mass index; WC: waist circumference; TC: total cholesterol; TG: triglyceride; HDL-C: high density lipoprotein cholesterol; LDL-C: low density lipoprotein cholesterol.

**Table 2:** Association of 28 SNPs tested with T2D in a Mongolian sample.

Chromosome	Nearby gene	SNP	Risk allele	Risk allele frequency		*P* value	OR	OR L95^a^	OR H95^b^
				Cases	Controls				
2	*IRS1 *	rs7578326	A	0.843	0.792	6.4*E* − 08	1.27	0.98	1.64
12	*HMGA2 *	rs1531343	C	0.111	0.106	6.2*E* − 07	1.08	0.79	1.49
15	*PRC1 *	rs8042680	A	0.953	0.916	5.6*E* − 06	1.32	0.86	2.00
2	*THADA *	rs7578597	T	0.989	0.980	8.0*E* − 06	1.92	0.76	4.76
9	*CDKN2 *	rs1333051	A	0.836	0.833	7.3*E* − 05	1.05	0.80	1.39
2	*TMEM163 *	rs6723108	T	0.997	0.980	7.9*E* − 05	7.69	2.00	33.33
11	*KCNQ1 *	rs2237897	C	0.676	0.585	8.7*E* − 05	1.39	1.11	1.72
11	*KCNQ1 *	rs163182	C	0.427	0.358	1.0*E* − 04	1.37	1.11	1.69
11	*MTNR1B *	rs1387153	T	0.420	0.358	2.7*E* − 04	1.17	0.95	1.44
2	*BCL11A *	rs243021	A	0.715	0.696	1.2*E* − 04	1.33	1.06	1.67
7	*PAX4 *	rs10229583	G	0.848	0.817	4.5*E* − 04	1.28	0.97	1.69

3	*IGF2BP2 *	rs4402960	T	0.320	0.274	5.0*E* − 02	1.21	0.97	1.50
2	*GRB14 *	rs3923113	A	0.847	0.812	1.7*E* − 02	1.20	0.93	1.56
13	*SPRY2 *	rs1359790	G	0.698	0.679	9.9*E* − 03	1.18	0.95	1.47
3	*ADAMTS9 *	rs4607103	C	0.594	0.566	1.2*E* − 01	1.11	0.88	1.39
11	*KCNJ11 *	rs5215	C	0.370	0.336	6.1*E* − 03	1.03	0.84	1.25
10	*HHEX-IDE *	rs5015480	C	0.223	0.219	5.7*E* − 03	1.01	0.80	1.29
10	*TCF7L2 *	rs7903146	T	0.053	0.061	1.1*E* − 01	1.01	0.66	1.57
11	*CENTD2 *	rs1552224	A	0.896	0.891	5.3*E* − 02	1.00	0.72	1.39
15	*RASGRP1 *	rs7403531	T	0.375	0.359	2.5*E* − 02	0.98	0.80	1.20
4	*WFS1 *	rs1801214	T	0.947	0.943	5.8*E* − 02	0.98	0.62	1.54
16	*FTO *	rs8050136	A	0.162	0.166	1.3*E* − 01	0.98	0.75	1.30
8	*TP53INP1 *	rs896854	T	0.351	0.389	3.1*E* − 02	0.93	0.76	1.15
12	*LGR5 *	rs7961581	C	0.219	0.239	6.1*E* − 02	0.92	0.72	1.17
10	*GRK5 *	rs10886471	C	0.755	0.774	2.2*E* − 02	0.86	0.67	1.10
6	*C6orf57 *	rs1048886	G	0.148	0.212	1.2*E* − 04	0.67	0.51	0.89
15	*ZFAND6 *	rs11634397	G	0.178	0.231	2.6*E* − 05	0.67	0.52	0.86
5	*ZBED3 *	rs4457053	G	0.055	0.090	1.4*E* − 02	0.63	0.43	0.94

^a^Lower boundary of 95% confidence interval (CI) of odd's ratio.

^b^Upper boundary of 95% CI of odd's ratio.

**Table 3:** Comparison of risk allele frequency of 28 SNPs tested for association with T2D in a Mongolian sample against 1000G populations.

Nearby gene	SNP	T2D risk allele	T2D risk allele frequency				(MGL − CEU)/CEU	*F*_ST_ with Mongolian sample			T2D association
			MGL^a^	CEU^b^	CHS^c^	CHB^d^		CEU	CHS	CHB	
*TMEM163 *	rs6723108	T	0.98	0.51	1.00	1.00	0.94	0.66	0.01	0.01	Yes
*C6orf57 *	rs1048886	G	0.21	0.14	0.06	0.10	0.55	0.01	0.07	0.04	No
*WFS1 *	rs1801214	T	0.94	0.65	0.88	0.98	0.46	0.34	0.03	0.01	No
*BCL11A *	rs243021	A	0.70	0.48	0.66	0.63	0.45	0.06	0.23	0.19	Yes
*MTNR1B *	rs1387153	T	0.36	0.25	0.45	0.41	0.42	0.02	0.02	0.00	Yes
*GRK5 *	rs10886471	C	0.77	0.55	0.76	0.79	0.42	0.21	0.00	0.00	No
*GRB14 *	rs3923113	A	0.81	0.59	0.89	0.81	0.37	0.12	0.02	0.00	No
*RASGRP1 *	rs7403531	T	0.36	0.28	0.29	0.34	0.29	0.01	0.01	0.00	No
*PRC1 *	rs8042680	A	0.92	0.72	1.00	1.00	0.28	0.67	0.05	0.04	Yes
*IRS1 *	rs7578326	A	0.79	0.66	0.83	0.86	0.20	0.04	0.00	0.01	Yes
*THADA *	rs7578597	T	0.98	0.88	0.99	1.00	0.12	0.13	0.00	0.00	Yes

*PAX4 *	rs10229583	G	0.82	0.75	0.84	0.84	0.09	0.01	0.00	0.00	Yes
*CENTD2 *	rs1552224	A	0.89	0.88	0.91	0.90	0.01	0.00	0.00	0.00	No
*HMGA2 *	rs1531343	C	0.11	0.11	0.12	0.11	0.00	0.00	0.00	0.00	Yes
*CDKN2 *	rs1333051	A	0.83	0.84	0.88	0.86	−0.01	0.00	0.00	0.00	Yes
*SPRY2 *	rs1359790	G	0.68	0.73	0.70	0.67	−0.07	0.00	0.00	0.00	No
*LGR5 *	rs7961581	C	0.24	0.26	0.21	0.19	−0.09	0.00	0.00	0.00	No
*TP53INP1 *	rs896854	T	0.39	0.43	0.30	0.31	−0.09	0.00	0.02	0.01	No

*IGF2BP2 *	rs4402960	T	0.27	0.31	0.23	0.25	−0.11	0.00	0.00	0.00	No
*KCNJ11 *	rs5215	C	0.34	0.38	0.40	0.38	−0.12	0.00	0.00	0.00	No
*KCNQ1 *	rs163182	C	0.36	0.41	0.31	0.39	−0.13	0.00	0.00	0.00	Yes
*ADAMTS9 *	rs4607103	C	0.57	0.78	0.64	0.57	−0.28	0.09	0.01	0.00	No
*ZFAND6 *	rs11634397	G	0.23	0.34	0.07	0.08	−0.33	0.33	0.07	0.06	No
*KCNQ1 *	rs2237897	C	0.58	0.94	0.66	0.62	−0.38	0.23	0.01	0.00	Yes
*FTO *	rs8050136	A	0.17	0.44	0.14	0.15	−0.63	0.19	0.00	0.00	No
*ZBED3 *	rs4457053	G	0.09	0.30	0.04	0.07	−0.70	0.17	0.01	0.00	No
*TCF7L2 *	rs7903146	T	0.06	0.31	0.03	0.02	−0.81	0.27	0.01	0.01	No

^a^Mongolian sample of this study. ^b,c,d^Population samples of Caucasian, Chinese in Beijing, and Chinese in Shanghai from the 1000G project, respectively.
