# Supplementary material for: Association Analysis of Genetic Variants with Type 2 Diabetes in a Mongolian Population in China
Source: J Diabetes Res. 2015 Jul 28;2015:613236. doi: 10.1155/2015/613236 (PMC4531200; doi:10.1155/2015/613236)
Supplement: Supplementary file 1 — The SNPs were chosen with following considerations: (1) GWAS SNPs found to be associated with T2D in an Asian sample were given higher priority; and (2) subsequently, SNPs found to be associated with T2D in multiple studies were included. Genotyping of six SNPs was not successful, thus these SNPs were included from further analysis. [file 613236.f1.pdf]

Supplemental table 1. 34 SNPs selected for testing with T2D in a Mongolian sample

				Reported in current study			Reported in original work			
Chromosome	Nearby gene	SNP	Risk allele	risk allele frequency	P value	OR	risk allele frequency	P value	OR	Reference
2	IRS1	rs7578326	A	0.843	6.40E-08	1.27	NR	5.00E-20	1.11	(Voight et al., 2010)
12	HMGA2	rs1531343	C	0.111	6.16E-07	1.08	NR	4.00E-09	1.1	(Voight et al., 2010)
15	PRC1	rs8042680	A	0.953	5.56E-06	1.32	NR	2.00E-10	1.07	(Voight et al., 2010)
2	THADA	rs7578597	T	0.989	8.01E-06	1.92	0.9	1.00E-09	1.15	(Zeggini et al., 2008)
9	CDKN2	rs1333051	A	0.836	7.25E-05	1.05	NR	6.00E-10	1.22	(Parra et al., 2011)
2	TMEM163	rs6723108	T	0.997	7.89E-05	7.69	0.86	7.00E-08	1.27	(Tabassum et al., 2013)
11	KCNQ1	rs2237897	C	0.676	8.66E-05	1.39	0.34	1.00E-16	1.33	(Unoki et al., 2008)
11	KCNQ1	rs163182	C	0.427	1.03E-04	1.37	0.34	2.00E-17	1.28	(Cui et al., 2011)
11	MTNR1B	rs1387153	T	0.420	2.71E-04	1.17	NR	8.00E-15	1.09	(Voight et al., 2010)
2	BCL11A	rs243021	A	0.715	1.21E-04	1.33	NR	3.00E-15	1.08	(Voight et al., 2010)
7	PAX4	rs10229583	G	0.848	4.54E-04	1.28	0.825	2.00E-10	1.14	(Ma et al., 2013)
3	IGF2BP2	rs4402960	T	0.320	0.049841	1.21	0.3	9.00E-16	1.14	(Takeuchi et al., 2009)
2	GRB14	rs3923113	A	0.847	0.017313	1.2	0.74	1.00E-08	1.09	(Kooner et al., 2011)
13	SPRY2	rs1359790	G	0.698	9.88E-03	1.18	0.71	6.00E-09	1.15	(Shu et al., 2010)
3	ADAMTS9	rs4607103	C	0.594	0.11849	1.11	0.76	1.00E-08	1.09	(Zeggini et al., 2008)
11	KCNJ11	rs5215	C	0.370	6.08E-03	1.03	NR	5.00E-11	1.14	(Zeggini et al., 2008)
10	HHEX,IDE	rs5015480	C	0.223	0.0056731	1.01	0.17	9.00E-06	1.17	(Shu et al., 2010)
10	TCF7L2	rs7903146	T	0.053	0.10591	1.01	0.18	1.00E-08	1.34	(Scott et al., 2007)
11	CENTD2	rs1552224	A	0.896	0.053174	1	NR	1.00E-22	1.14	(Voight et al., 2010)
15	RASGRP1	rs7403531	T	0.375	2.46E-02	0.98	0.346	4.00E-09	1.1	(Li et al., 2013)
4	WFS1	rs1801214	T	0.947	0.058202	0.98	NR	3.00E-08	1.13	(Voight et al., 2010)
16	FTO	rs8050136	A	0.162	0.1277	0.98	0.38	1.00E-12	1.17	(Scott et al., 2007)
8	TP53INP1	rs896854	T	0.351	0.031176	0.93	NR	1.00E-09	1.06	(Voight et al., 2010)
12	LGR5	rs7961581	C	0.219	0.061213	0.92	0.27	1.00E-09	1.09	(Zeggini et al., 2008)
10	GRK5	rs10886471	C	0.755	0.021593	0.86	0.778	7.00E-09	1.12	(Li et al., 2013)
6	C6orf57	rs1048886	G	0.148	1.24E-04	0.67	0.18	3.00E-08	1.54	(Sim et al., 2011)
15	ZFAND6	rs11634397	G	0.178	2.59E-05	0.67	NR	2.00E-09	1.06	(Voight et al., 2010)
5	ZBED3	rs4457053	G	0.055	0.014219	0.63	NR	3.00E-12	1.08	(Voight et al., 2010)
8	ANK1	rs515071	?	failed			0.794	1.00E-08	1.18	(Imamura et al., 2012)
9	*CDKN2A,CDKN2B*	rs10811661	T	failed			0.85	8.00E-15	1.2	(Scott et al., 2007)
X	DUSP9	rs5945326	A	failed			0.606	7.00E-16	1.18	(Li et al., 2013)
7	CREB5,JAZF1	rs864745	T	failed			0.5	5.00E-14	1.1	(Zeggini et al., 2008)
9	PTPRD	rs17584499	T	failed			0.06	9.00E-10	1.57	(Tsai et al., 2010)
2	BCL11A	rs243021	A	failed			NR	3.00E-15	1.08	(Voight et al., 2010)
17	SRR	rs391300	G	failed			0.62	3.00E-09	1.28	(Tsai et al., 2010)
